# Vena cava filter placement during pregnancy: a therapeutic challenge

**DOI:** 10.1590/1677-5449.202600712

**Published:** 2026-10-09

**Authors:** Mateus Alves Borges Cristino, Ronald Luiz Gomes Flumignam, Gustavo Braga Murta, Marcos Arêas Marques

**Affiliations:** 1 Rede Mater Dei de Saúde, Belo Horizonte, MG, Brasil.; 2 Universidade Federal de São Paulo – UNIFESP, São Paulo, SP, Brasil.; 3 Universidade Federal do Estado do Rio de Janeiro – UNIRIO, Rio de Janeiro, RJ, Brasil.; 4 Universidade do Estado do Rio de Janeiro – UERJ, Rio de Janeiro, RJ, Brasil.

**Keywords:** pregnancy, high-risk, venous thrombosis, pulmonary embolism, vena cava filters, postpartum period, anticoagulants

## Abstract

Pulmonary embolism (PE) is the leading cause of maternal death during the antepartum-postpartum cycle in developed countries. The diagnosis of venous thromboembolism during pregnancy is complicated by the physiological changes that occur during gestation. Management with anticoagulants is the treatment of choice, while endovascular interventions are reserved for exceptional cases. We report a case of placement of a removable inferior vena cava filter (IVCF) in a pregnant woman with left iliofemoral venous thrombosis during latent labor. After delivery, the IVCF was successfully removed with no maternal or fetal complications and anticoagulation was resumed. Considering the risks associated with IVCFs in pregnant women, their use should be restricted to cases with formal contraindications to anticoagulation and high risk of PE. Additional precautions minimize fetal radiation exposure. The decision to place an IVCF during pregnancy is complex and requires interdisciplinary discussion. Further research is needed to assess its impact.

## INTRODUCTION

Pulmonary embolism (PE) is recognized as the number one cause of death during the antepartum-postpartum cycle in developed countries.^1^ All elements of Virchow’s triad are present during this cycle — venous stasis, hypercoagulability, and endothelial injury — and remain present up to the sixth week postpartum, elevating the risk of lower limb (LL) venous thromboembolism (VTE) by up to five times compared with the general population.^1-3^

It is a challenge to diagnose LL VTE in pregnant women, because signs and symptoms such as edema and pain are common during pregnancy. Additionally, the accuracy of tests decreases as gestation progresses, compromising early diagnosis. When VTE is diagnosed, the first-choice treatment is anticoagulation, reserving endovascular interventions such as inferior vena cava filter (IVCF) placement for exceptional circumstances. Use of an IVCF in a pregnant woman was first reported in 1981.^4^

We report a case in which a removable IVCF was placed in an expectant mother who developed deep venous thrombosis (DVT) in the left iliofemoral venous segment while in latent labor. In view of the risk of peripartum hemorrhage, the management approach chosen was temporary suspension of anticoagulation and placement of an IVCF as a prophylactic measure against pulmonary embolism.

This therapeutic challenge was approved by the Research Ethics Committee at the Hospital Mater Dei S/A, with Ethics Appraisal Submission Certificate 86787825.7.0000.5128 and Substantiated Opinion number 7.709.303.

### Part 1 – Clinical situation

A 33-year-old patient, in the 37th week of her first gestation, presented at the obstetric emergency room complaining of pain in the left lower limb with diffuse edema, calf tenderness and induration, and presence of prominent sentinel veins. Color Doppler ultrasound (CDU) of the LL venous system revealed signs of recent occlusive DVT in the left external iliac, common femoral, and femoral veins (Figures 1 [Fig gf0200] to 3). She was started on full anticoagulation with enoxaparin (60 mg, subcutaneous, twice a day). However, the patient was in latent labor, raising the issue of whether to suspend anticoagulation temporarily to mitigate the risk of hemorrhage during birth. In parallel, the possibility of placing an IVCF as a prophylactic measure against PE was also considered.

**Figure 1:**
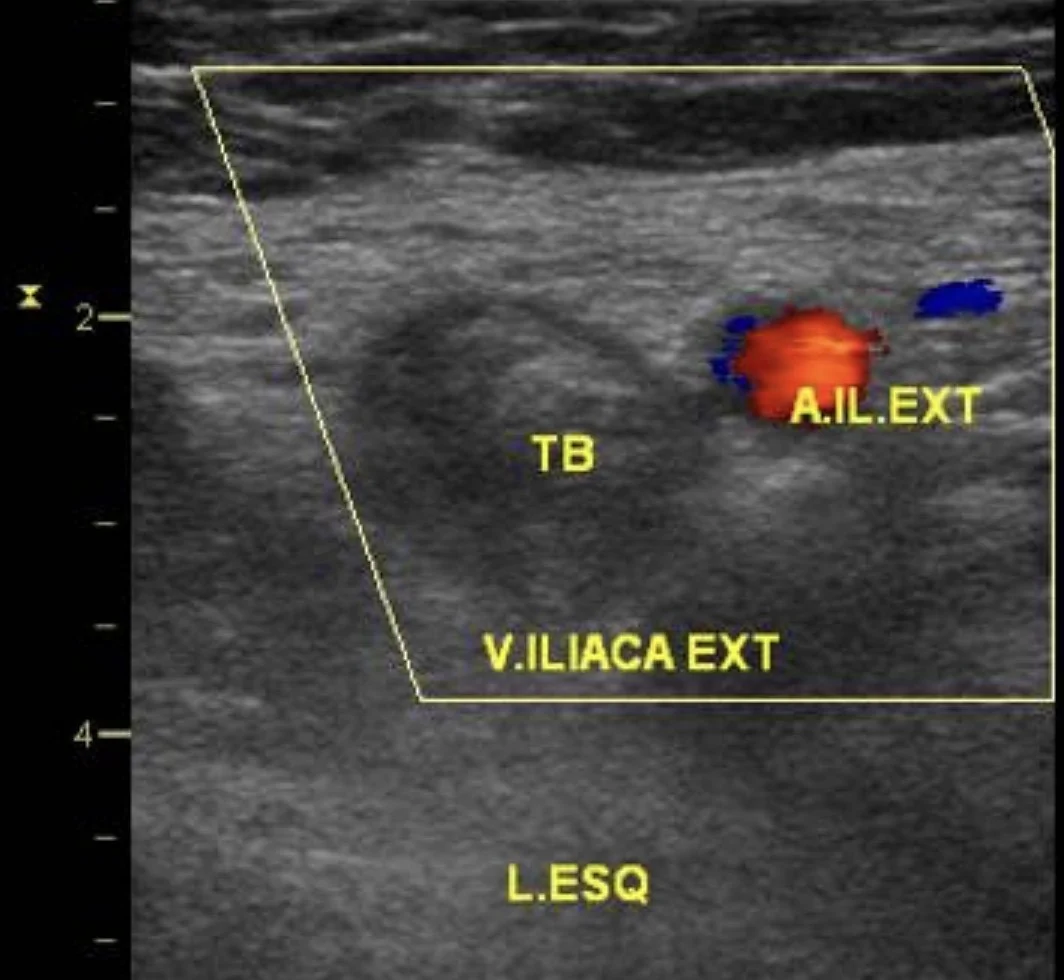
Color Doppler ultrasound images of the venous system, suggestive of recent occlusive deep venous thrombosis in the left external iliac vein. A.IL.EXT = external iliac artery; TB = thrombus; V.ILIACA EXT = external iliac vein; L.ESQ = left side.

**Figure 2 gf0200:**
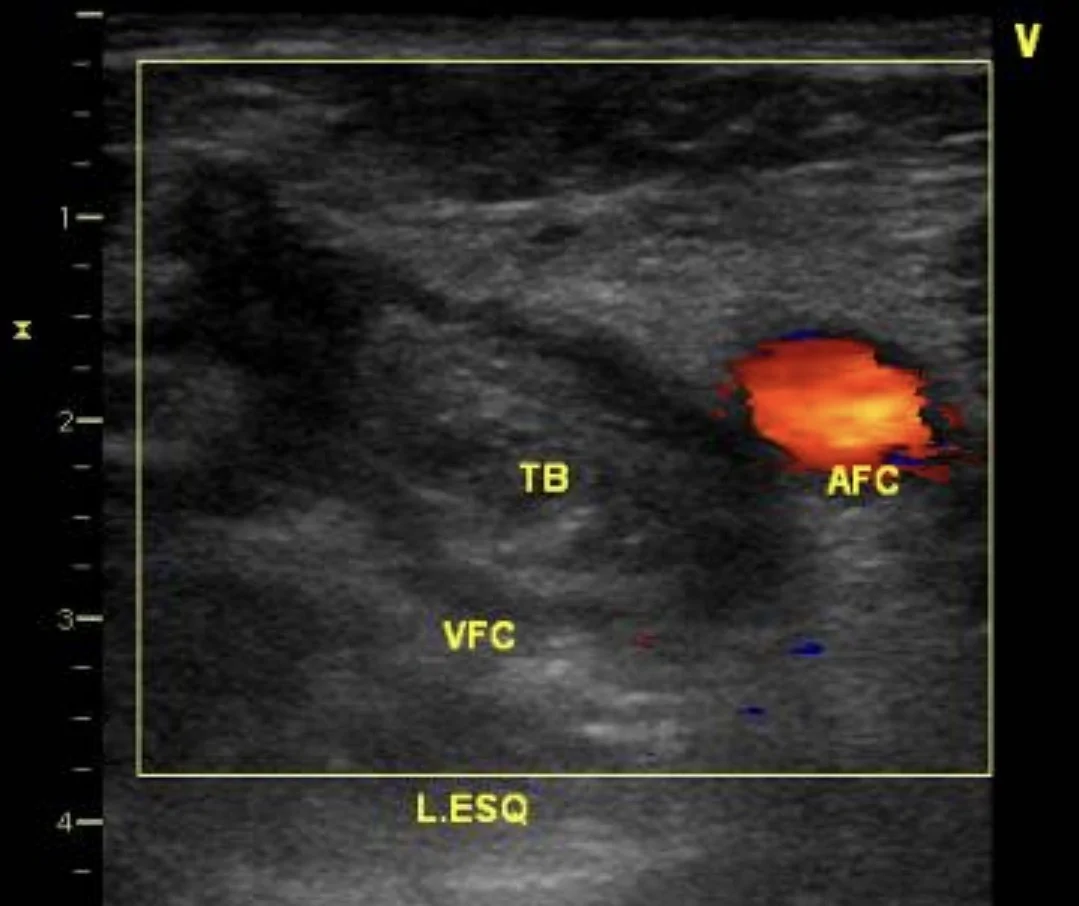
Color Doppler ultrasound images of the venous system suggestive of recent occlusive deep venous thrombosis in the left common femoral vein. TB = thrombus; AFC = common femoral artery; VFC = common femoral vein; L.ESQ = left side.

**Figure 3:**
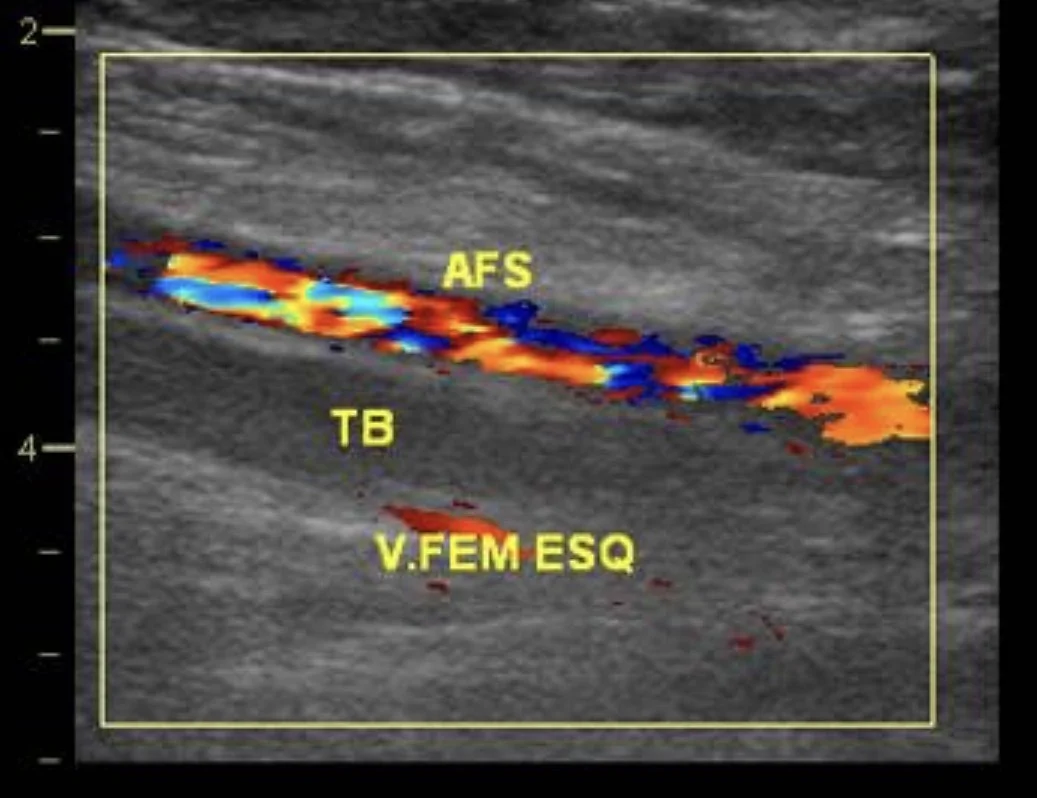
Color Doppler ultrasound images of the venous system suggestive of recent occlusive deep venous thrombosis in the left femoral vein. AFS = superficial femoral artery; TB = thrombus; V.FEM ESQ = left femoral vein.

### Part 2 – What was done

The decision was taken to fit a removable IVCF (Optease, Cordis®). In order to minimize radiation exposure, the route chosen was via the right internal jugular vein, using a lead shield to protect the abdominal area. The procedure was conducted under local anesthesia, with ultrasound-guided puncture and insertion of a 5 Fr x 11 cm introducer, followed by a 0.035” x 260 cm guidewire, before advancing a pigtail catheter down to the inferior vena cava and placing the suprarenal IVCF, above the fetal position (Figure 4). Control angiography showed the filter had been correctly positioned, with no signs of displacement or contrast leakage (Figure 5). After placement of the filter, anticoagulation was suspended and vaginal delivery was induced, under epidural anesthesia, with no maternal or fetal complications.

**Figure 4:**
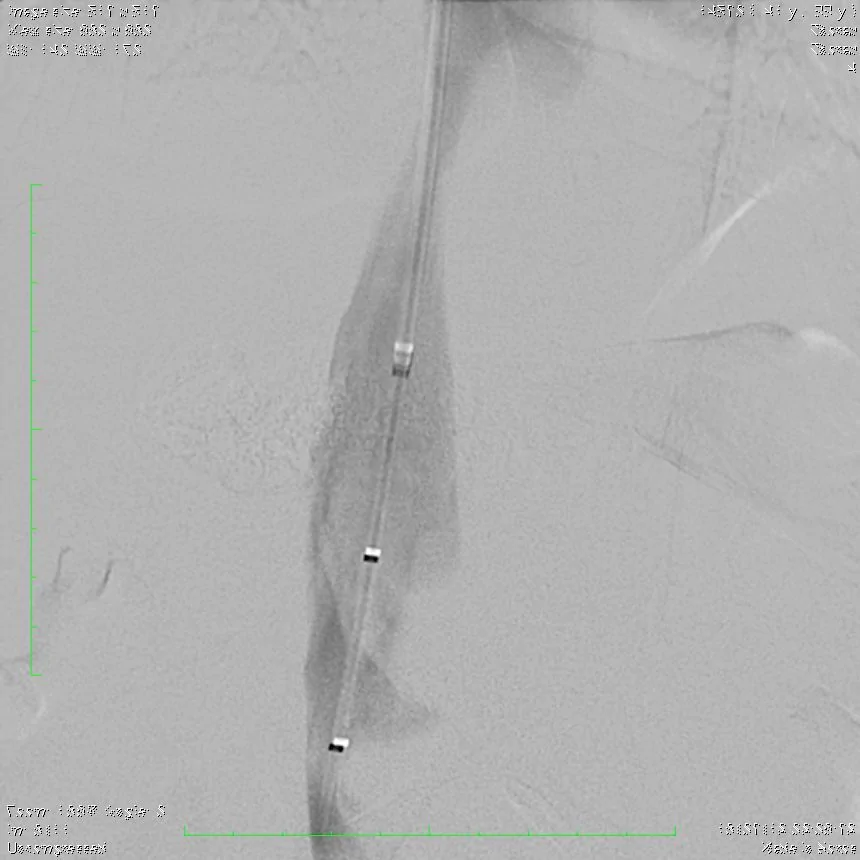
Placement of the inferior vena cava filter in the suprarenal area before release.

**Figure 5:**
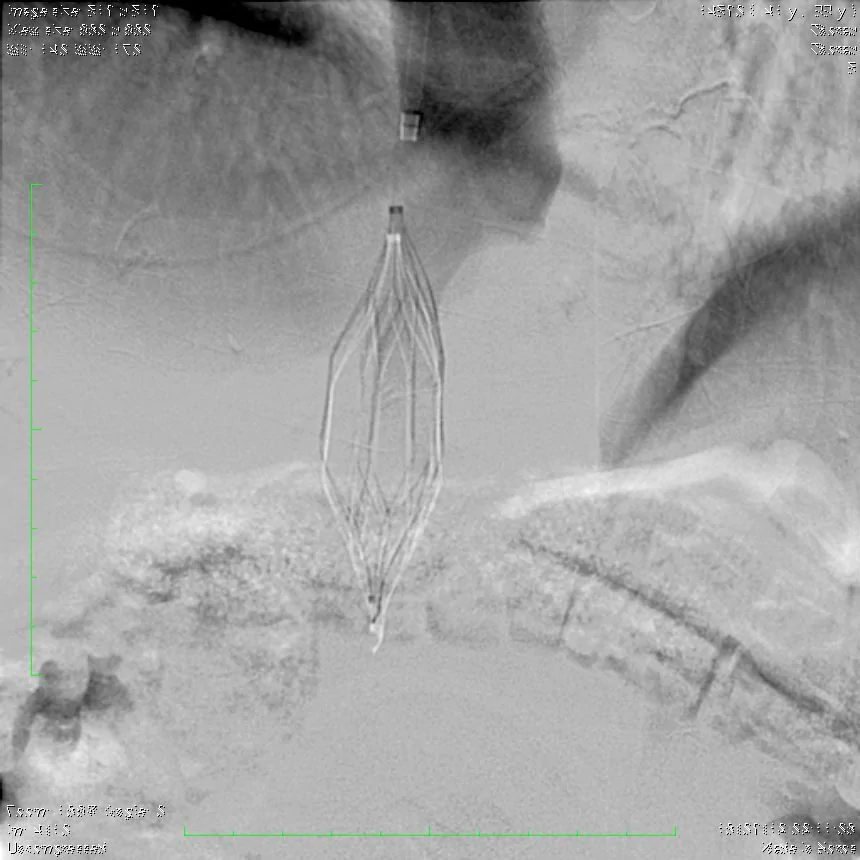
Control angiography showing the inferior vena cava filter correctly placed.

Twelve hours after delivery, full anticoagulation was resumed and, 48 hours later, the IVCF was removed via the right femoral vein, using a femoral introducer and 10 Fr guide catheter. After an unsuccessful attempt using a single loop snare catheter, an Indy (Cook^®^) multi-loop snare catheter was used to successfully remove the filter (Figures 6 and 7**)**. The patient was discharged from hospital on the second postoperative day, on enoxaparin (60 mg, subcutaneous, twice a day) and wearing elastic compression stockings, transitioning to oral warfarin during the 6-month lactation period.

**Figure 6:**
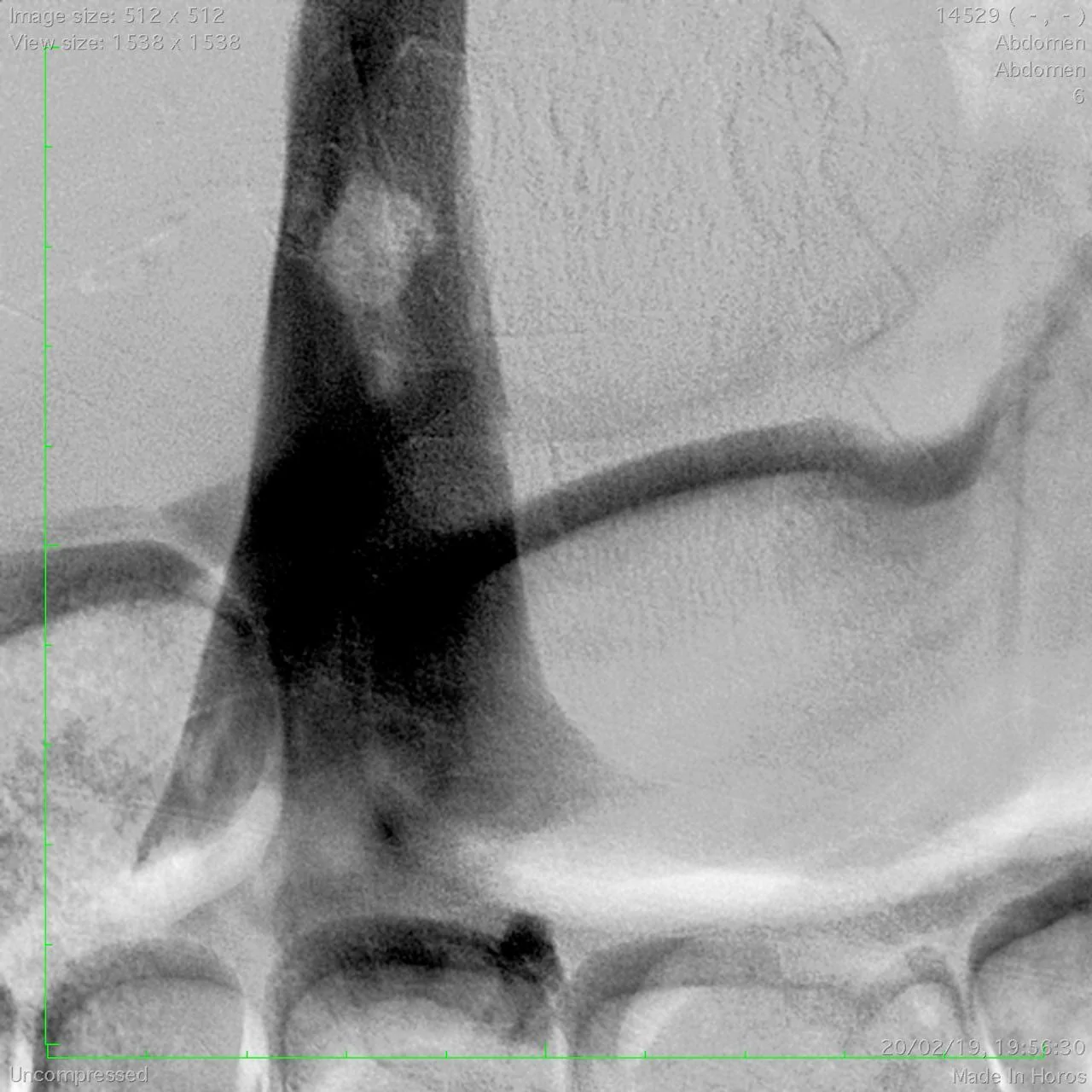
Angiography of the inferior vena cava after removal of the inferior vena cava filter, showing it is intact, with well-defined margins, and free from contrast leakage.

**Figure 7:**
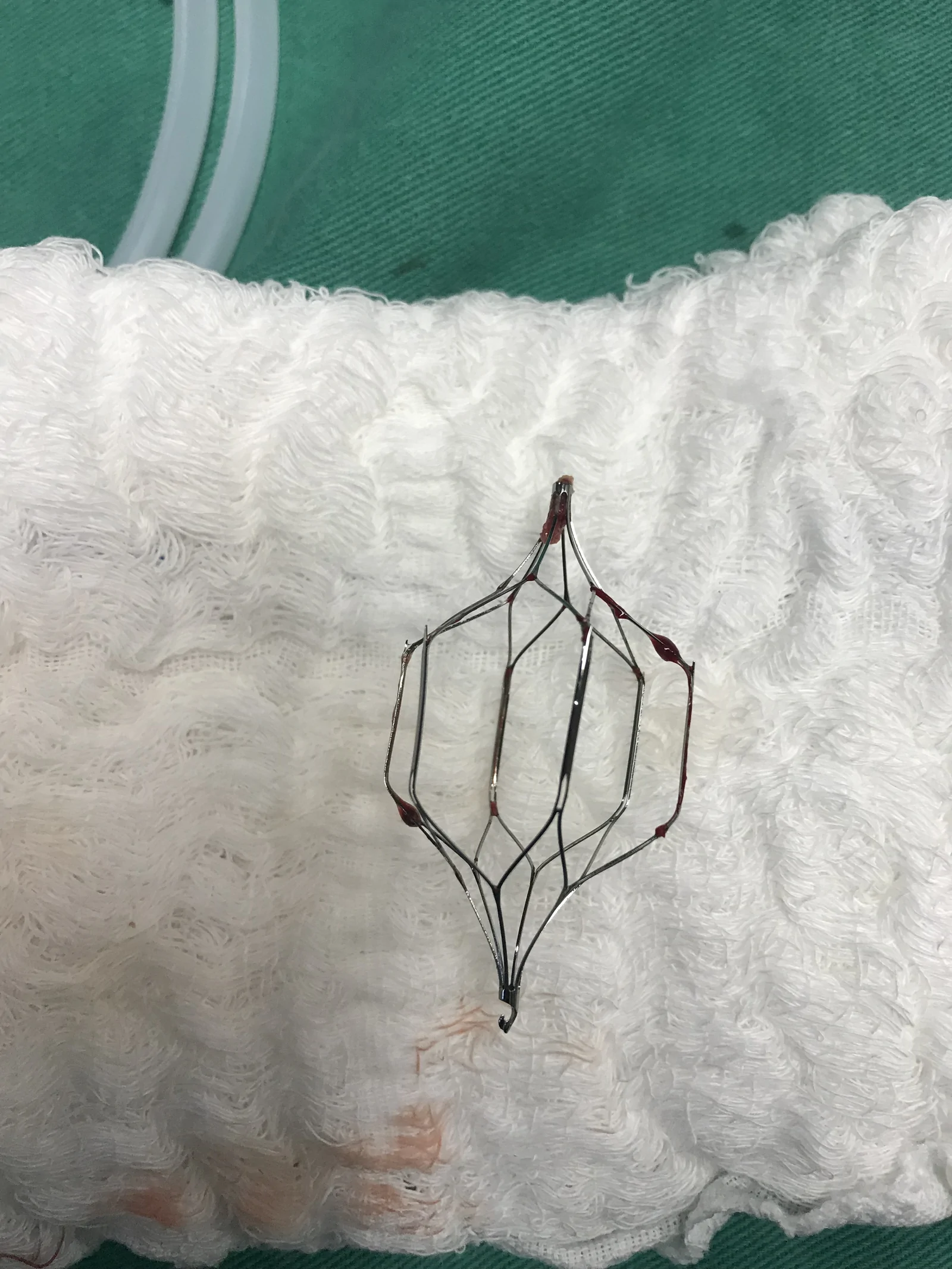
Inferior vena cava filter successfully removed 48 hours after placement.

The patient has been in regular outpatient follow-up for 7 years, with annual CDU scans to rule out postthrombotic syndrome (PTS). At her last consultation, she was at clinical stage C1 of the Clinical, Etiology, Anatomy, Pathophysiology (CEAP) classification, asymptomatic, and at Villalta PTS score of 0.

## DISCUSSION

Diagnosis of VTE during the antepartum-postpartum cycle is hindered by certain challenges. Signs and symptoms such as edema and pain can be confused with physiological changes that are typical of pregnancy.^1,2^ Isolated DVT in the iliac, which is the most common presentation in this phase, can manifest with abdominal or lumbar pains, both common symptoms during gestation.^5^

All pregnant women with signs and symptoms suggestive of VTE should be started on full anticoagulation, unless there are contraindications, until the diagnosis is confirmed or ruled out.^1,2^ Of the available supplementary imaging methods, CDU and magnetic resonance angiography (MRIa) stand out. Angiotomography can be considered an alternative to MRIa for assessing the pelvic area, but it should be avoided whenever possible, because it exposes the fetus to radiation.^6^

Color Doppler ultrasound, with application of venous compression using the transducer, is a noninvasive, low-cost method, with high sensitivity and specificity for diagnosis of femoropopliteal DVT.^5^ However, it has limitations in the iliocaval segment because of the depth of the vessels and because of the gravid uterus.^7^

Although endovascular materials and devices have improved, placement of an IVCF is not free from risks, such as insertion-related trauma, migration, fracture, endothelization, perforation of the vena cava, or DVT. The decision to place an IVCF during pregnancy is complex, but it should be considered in situations in which anticoagulation is contraindicated because of the risk of hemorrhage during delivery and fetal radiation exposure.

The guidelines of the American College of Chest Physicians^8^ and the American College of Obstetricians and Gynecologists^9^ do not contain specific recommendations for this situation. However, the Royal College of Obstetricians and Gynaecologists has published guidelines recommending that a removable IVCF should be considered during the peripartum period for patients with iliac DVT or recurrent PE despite adequate anticoagulation.^10^ The Society of Interventional Radiology recognizes absolute and relative indications for placement of an IVCF in cases of confirmed VTE and for PE prophylaxis.^11^

Harris et al.^12^ conducted a systematic review, in which they observed that the only reason for placement of an IVCF was the presence of extensive DVT during gestation and the risk of PE during the peripartum period, when anticoagulation should be suspended. This was particularly evident in cases with iliofemoral DVT and birth imminent within the next 2 or 3 weeks.^13^

In a review article, Crosby et al.^14^ analyzed 40 pregnant women who had had a removable IVCF fitted, totaling 43 procedures, and compared the data with a review of the literature on IVCF placement in women who were not pregnant. There were no cases of PE among the pregnant women (0/43), whereas 57 cases were observed in 6,291 women who were not pregnant (0.9%). Complications such as thrombosis of the IVCF (2.3% vs. 0.9%; p = 0.33) and perforation of the inferior vena cava (7.0% vs. 4.4%; p = 0.44) were more common among the pregnant women, but without statistical significance. Unsuccessful attempts to remove filters were more common in pregnant women (26% vs. 11%; p = 0.006) and were not correlated with type of device, time since placement, or type of delivery.

In another systematic review, Harris et al.^12^ analyzed publications from 1981 to 2014, finding 124 cases of IVCF placement in pregnant women. There were no recorded cases of PE, but there was one maternal death during placement of a filter, caused by air embolism. Complications related to the IVCF were observed in 8.8% of cases, whereas incidence in the general population is 0.3%.^15^

### Considerations on the placement technique

Successful IVCF placement in pregnant women is described in all trimesters of gestation (from week 7 to week 41),^16^ including during latent labor.^17^ The internal jugular route is preferred, to minimize radiation exposure, although femoral access is also feasible.^18,19^ In cases in which the vena cava is compressed by the gravid uterus, putting the patient in a lateral recumbent position can facilitate the procedure.^17^

A suprarenal IVCF position can offer additional benefits, such as protection from PE originating in dilated ovarian veins, and greater blood flow through the filter interior, reducing venous stasis.^18,19^

### Fetal morbidity and exposure to radiation

To minimize fetal radiation exposure, especially from the 8th to the 15th weeks, measures such as positioning the uterus outside of the radiological field, appropriate collimation, and use of lead shielding are essential.^20^ Use of intravascular ultrasound can reduce exposure to radiation, but its efficacy in pregnant women is still a matter of debate.^20^

### Use of iodinated contrast in gestation

Iodinated contrast can pass through the placental barrier, but studies in animals and humans have not demonstrated teratogenic or mutagenic effects.^20^ Suspension of breastfeeding after use of contrast is no longer recommended, because less than 1% of the administered dose is excreted into breast milk.^21^

### Other complications

If the IVCF remains in place for a prolonged period, complications may occur, such as fracture with embolism of fragments, penetration of the inferior vena cava wall, and thrombosis of the vena cava.^22^

## CONCLUSIONS

Venous thromboembolism during gestation is a complex condition that demands case-by-case management. Placement of a removable IVCF may be a safe and effective option in some situations, such as when anticoagulation is temporarily contraindicated.

## DISPONIBILIDADE DE DADOS

Os dados que sustentam este estudo estão disponíveis mediante solicitação ao autor correspondente, MABC. Devido a questões éticas e de confidencialidade, os dados não podem ser disponibilizados publicamente.
