# Supplementary material for: Vena cava filter placement during pregnancy: a therapeutic challenge
Source: J Vasc Bras. 2026 Oct 9;25:e20260071. doi: 10.1590/1677-5449.202600712 (PMC13670994; doi:10.1590/1677-5449.202600712)
Supplement: Translation

# Translation: Implante de filtro de veia cava em gestante: desafio terapêutico

**DOI:** 10.1590/1677-5449.202600711

**Authors:** Mateus Alves Borges Cristino, Ronald Luiz Gomes Flumignam, Gustavo Braga Murta, Marcos Arêas Marques

**Affiliations:** 1 Rede Mater Dei de Saúde, Belo Horizonte, MG, Brasil.; 2 Universidade Federal de São Paulo – UNIFESP, São Paulo, SP, Brasil.; 3 Universidade Federal do Estado do Rio de Janeiro – UNIRIO, Rio de Janeiro, RJ, Brasil.; 4 Universidade do Estado do Rio de Janeiro – UERJ, Rio de Janeiro, RJ, Brasil.

**Keywords:** gravidez de alto risco, trombose venosa profunda, embolia pulmonar, filtros de veia cava, período pós-parto, anticoagulantes

## Abstract

A embolia pulmonar é a principal causa de morte materna durante o ciclo gravídico-puerperal em países desenvolvidos. O diagnóstico do tromboembolismo venoso na gravidez é dificultado pelas mudanças fisiológicas que ocorrem na gestação. O tratamento com anticoagulantes é a abordagem de escolha, sendo intervenções endovasculares procedimentos de exceção. Relatamos um caso de implante de filtro de veia cava inferior removível em gestante com trombose venosa ilíaco-femoral esquerda em período de parto latente. Após o parto, o filtro de veia cava inferior foi removido com sucesso, sem complicações maternas ou fetais, e a anticoagulação foi retomada. Considerando os riscos associados ao filtro de veia cava inferior em gestantes, seu uso deve ser restrito a casos com contraindicações formais à anticoagulação e alto risco de embolia pulmonar. Precauções adicionais minimizam a exposição fetal à radiação. A decisão pelo filtro de veia cava inferior na gravidez é complexa, requer discussão interdisciplinar e, além disso, pesquisas adicionais são necessárias para avaliar seu impacto.

## INTRODUÇÃO

A embolia pulmonar (EP) é reconhecida como a principal causa de morte durante o ciclo gravídico-puerperal nos países desenvolvidos^1^ . Durante esse ciclo, a tríade de Virchow, composta por estase venosa, hipercoagulabilidade e lesão endotelial, está integralmente presente, persistindo até a sexta semana do período pós-parto, elevando o risco de tromboembolismo venoso (TEV) de membros inferiores (MMII) em até cinco vezes em comparação à população geral^1-3^ .

O diagnóstico de TEV de MMII em gestantes é desafiador, uma vez que sinais e sintomas como edema e dor são frequentes durante a gravidez. Além disso, a acurácia dos exames complementares diminui com a progressão da gestação, dificultando o diagnóstico precoce. Após o diagnóstico de TEV, a anticoagulação é a primeira linha terapêutica, enquanto intervenções endovasculares, como o implante de filtro de veia cava inferior (FVCI), são reservadas para situações de exceção. O primeiro relato de uso de FVCI em gestantes remonta a 1981^4^ .

Relatamos um caso de implante de FVCI removível em uma gestante que, em trabalho de parto latente, apresentou trombose venosa profunda (TVP) ilíaco-femoral à esquerda. Diante do risco de hemorragia periparto, optou-se pela suspensão temporária da anticoagulação e pela inserção do FVCI como medida profilática para embolia pulmonar.

Este desafio terapêutico foi aprovado pelo Comitê de Ética em Pesquisa do Hospital Mater Dei S/A sob o Certificado de Apresentação para Apreciação Ética: 86787825.7.0000.5128 e o Parecer nº 7.709.303.

### Parte 1 – Situação clínica

Paciente primigesta, 33 anos de idade, na 37ª semana de gestação, compareceu ao pronto-socorro obstétrico com queixa de dor no membro inferior esquerdo, associada a edema difuso, empastamento da panturrilha e presença de veias sentinelas. O eco-Doppler colorido (EDC) venoso dos MMII revelou sinais de TVP recente e oclusiva na ilíaca externa, na femoral comum e na femoral esquerda (Figuras 1 [Fig gf02] a 3). Iniciou-se anticoagulação plena com enoxaparina (60 mg, subcutânea, duas vezes ao dia). No entanto, a gestante encontrava-se em trabalho de parto latente, o que levantou a questão da suspensão temporária da anticoagulação para mitigar o risco de hemorragia durante o parto. Paralelamente, considerou-se o implante de FVCI como medida profilática contra a EP.

**Figura 1:** Eco-Doppler colorido venoso sugestivo de trombose venosa profunda recente e oclusiva de veia ilíaca externa esquerda. A.IL.EXT = artéria ilíaca externa; TB = trombo; V.ILIACA EXT = veia ilíaca externa; L.ESQ = lado esquerdo.

**Figura 2 gf02:** Eco-Doppler colorido venoso sugestivo de trombose venosa profunda recente e oclusiva de veia femoral comum esquerda. TB = trombo; AFC = artéria femoral comum; VFC = veia femoral comum; L.ESQ = lado esquerdo.

**Figura 3:** Eco-Doppler colorido venoso sugestivo de trombose venosa profunda recente e oclusiva de veia femoral esquerda. AFS = artéria femoral superficial; TB = trombo; V.FEM ESQ = veia femoral esquerda.

### Parte 2 – O que foi feito

Optou-se pelo implante de FVCI removível (Optease, Cordis®). Para reduzir a exposição à radiação, a via escolhida foi a veia jugular interna direita, com proteção abdominal com manta plúmbica. O procedimento foi realizado sob anestesia local, com punção ecoguiada e implante de introdutor 5 Fr x 11 cm, passagem de fio-guia 0,035” x 260 cm, descida de cateter *pigtail* para veia cava inferior e implante do FVCI suprarrenal, acima da posição fetal (Figura 4). A angiografia de controle evidenciou filtro bem posicionado, sem deslocamento ou extravasamento de contraste (Figura 5). Após o implante, a anticoagulação foi suspensa e o parto induzido por via vaginal, sob anestesia peridural, transcorrendo sem intercorrências maternas ou fetais.

**Figura 4:** Posicionamento do filtro de veia cava inferior em topografia suprarrenal antes de sua liberação.

**Figura 5:** Angiografia de controle com o filtro de veia cava inferior bem posicionado.

Após 12 horas do parto, a anticoagulação plena foi retomada e, 48 horas depois, o FVCI foi removido por via femoral direita, com introdutor femoral e cateter-guia 10 Fr e, após tentativa sem sucesso com cateter *snare* monolaço, optou-se pelo laço Indy (Cook^®^), que permitiu a sua remoção bem-sucedida (Figuras 6 e 7**)**. A paciente recebeu alta hospitalar no segundo dia pós-operatório, com enoxaparina (60 mg, subcutânea, duas vezes ao dia) e meias de compressão elástica, com posterior transição para varfarina oral durante o período de lactação de 6 meses.

**Figura 6:** Angiografia de veia cava inferior após a remoção do filtro de veia cava inferior, evidenciando sua integridade, com limites bem definidos e ausência de extravasamento de contraste.

**Figura 7:** Filtro de veia cava inferior removido com sucesso 48 horas após o seu implante.

A paciente permanece em acompanhamento regular ambulatorial há 7 anos, com EDC anual para avaliação da síndrome pós-trombótica (SPT). Na última consulta, apresentava-se em estágio clínico C1 da classificação CEAP (acrônimo do inglês, *Clinical, Etiology, Anatomy, Pathophysiology*) e assintomática, escore de Villalta para SPT igual a zero.

## DISCUSSÃO

O diagnóstico de TEV durante o ciclo gravídico-puerperal apresenta desafios peculiares. Sinais e sintomas como edema e dor podem ser confundidos com alterações fisiológicas típicas da gravidez^1,2^ . A TVP isolada da veia ilíaca, mais frequente nessa fase, pode manifestar-se com dor abdominal ou lombar, sintomas frequentes na gestação^5^ .

Todas as gestantes com sinais e sintomas sugestivos de TEV devem iniciar anticoagulação plena, a menos que haja contraindicação, até que o diagnóstico seja confirmado ou descartado^1,2^ . Entre os métodos complementares disponíveis, destacam-se o EDC e a angiorressonância nuclear magnética (aRNM). A angiotomografia deve ser considerada uma alternativa à aRNM para avaliação do segmento pélvico, mas sua utilização deve ser evitada sempre que possível devido à exposição fetal à radiação^6^ .

O EDC, com compressão venosa pelo transdutor, é um método não invasivo, de baixo custo e com alta sensibilidade e especificidade para o diagnóstico de TVP femoropoplítea^5^ . No entanto, sua limitação no segmento cavo-ilíaco deve-se à profundidade dos vasos e às limitações impostas pelo útero gravídico^7^ .

Apesar dos avanços nos materiais e dispositivos endovasculares, o implante do FVCI não é isento de riscos, como traumatismo durante sua inserção, migração, fratura, endotelização, perfuração da veia cava ou TVP. A decisão de implantar um FVCI durante a gestação é complexa, devendo ser considerada em situações em que a anticoagulação é contraindicada devido ao risco de hemorragia durante o parto e à exposição fetal à radiação.

As diretrizes do American College of Chest Physicians^8^ e do American College of Obstetricians and Gynecologists^9^ não possuem orientações específicas para essa situação. No entanto, as diretrizes publicadas pelo Royal College of Obstetricians and Gynaecologists recomendam considerar o uso de FVCI removível no período periparto para pacientes com TVP ilíaca ou EP recorrente, apesar da anticoagulação adequada^10^ . A Society of Interventional Radiology reconhece as indicações absolutas e relativas para a inserção de FVCI no TEV confirmado e para a profilaxia da EP^11^ .

Em revisão sistemática, Harris et al.^12^ observaram que a única razão para o implante de FVCI foi a presença de TVP extensa durante a gestação e o risco de EP no período periparto, quando a anticoagulação deveria ser suspensa. Isso foi particularmente evidente em casos de TVP ilíaco-femoral com parto iminente nas 2 ou 3 semanas seguintes^13^ .

Em um artigo de revisão, Crosby et al.^14^ analisaram 40 gestantes submetidas a implante de FVCI removível, totalizando 43 procedimentos, e compararam os dados com uma revisão da literatura com implante em não gestantes. Nenhum caso de EP foi registrado no grupo das gestantes (0/43), enquanto 57 casos foram observados em 6.291 não gestantes (0,9%). Complicações como trombose do FVCI (2,3% *versus* 0,9%; p = 0,33) e perfuração da veia cava inferior (7,0% *versus* 4,4%; p = 0,44) foram mais comuns nas gestantes, mas sem significância estatística. O insucesso na remoção do filtro foi mais frequente nas gestantes (26% *versus* 11%; p = 0,006) e não se correlacionou com o tipo de dispositivo, o tempo de inserção ou a via de parto.

Em outra revisão sistemática, Harris et al.^12^ analisaram publicações entre 1981 e 2014, encontrando 124 casos de FVCI implantados em gestantes. Nenhum caso de EP foi registrado, e um óbito materno ocorreu durante a inserção do filtro, por embolia aérea. Complicações relacionadas ao FVCI foram observadas em 8,8% dos casos, enquanto na população geral a incidência é de 0,3%^15^ .

### Considerações sobre a técnica de implante

O implante de FVCI em gestantes é descrito com sucesso em diferentes trimestres da gestação (da 7ª à 41ª semana)^16^ , inclusive em trabalho de parto latente^17^ . A via jugular interna é preferida para minimizar a exposição à radiação, embora a via femoral também seja viável^18,19^ . Em casos de compressão da veia cava pelo útero gravídico, o posicionamento da paciente em decúbito lateral pode facilitar o procedimento^17^ .

A posição suprarrenal do FVCI pode oferecer benefícios adicionais, como proteção contra EP originária de veias ovarianas dilatadas e maior fluxo sanguíneo no interior do filtro, reduzindo a estase venosa^18,19^ .

### Morbidade fetal e exposição à radiação

Para minimizar a exposição fetal à radiação, especialmente entre a 8ª e a 15ª semana, medidas como posicionamento do útero fora do campo radiológico, colimação adequada e uso de proteção plúmbica são essenciais^20^ . O uso de ultrassom intravascular pode reduzir a exposição à radiação, mas sua viabilidade em gestantes ainda é questionável^20^ .

### Uso de contraste iodado na gestação

O contraste iodado pode atravessar a barreira placentária, mas estudos em animais e humanos não demonstraram efeitos teratogênicos ou mutagênicos^20^ . A suspensão da amamentação após o uso de contraste não é mais recomendada, pois menos de 1% do contraste é excretado no leite materno^21^ .

### Outras complicações

A permanência prolongada do FVCI pode ocasionar complicações, como fratura com embolia do fragmento, penetração na parede da veia cava inferior e trombose da veia cava^22^ .

## CONCLUSÃO

O TEV durante a gestação é uma condição complexa que exige abordagem individualizada. O implante de FVCI removível pode ser uma opção segura e eficaz em algumas situações, como nos casos de contraindicação temporária à anticoagulação.

## Data Availability

The data supporting this study are available from the corresponding author, MABC. Due to ethical and confidentiality concerns, the data cannot be made publicly available.
